# Nonlinear relationship between gonadotropin total dose applied and live birth rates in non-PCOS patients: a retrospective cohort study

**DOI:** 10.1038/s41598-024-51991-y

**Published:** 2024-01-17

**Authors:** Xiaoyuan Xu, Aimin Yang, Yan Han, Siran Li, Wei Wang, Guimin Hao, Na Cui

**Affiliations:** Hebei Key Laboratory of Infertility and Genetics, Department of Reproductive Medicine, Hebei Clinical Research Center for Birth Defects, Second Hospital of Hebei Medical University, Shijiazhuang, 050000 China

**Keywords:** Endocrine reproductive disorders, Pregnancy outcome

## Abstract

The purpose of this article is to explore the relationship between the total dose of follicle-stimulating hormone (FSH) applied during controlled ovulation stimulation and the live birth rates (LBRs) in non-PCOS population. Many studies have found no difference between the dose of FSH application and pregnancy outcomes such as clinical pregnancy rates after fresh embryo transfer. However, a recent large retrospective analysis found a negative correlation between live birth rates and increasing dose of FSH. It is still controversial about the association between FSH dose and LBRs. In addition, no studies have yet explored the nonlinear relationship between FSH and LBRs. This cohort study included a total of 11,645 patients who had accepted IVF/intracytoplasmic sperm injection (ICSI) at the second hospital of Hebei medical university between December 2014 to December 2019. PCOS was identified by Rotterdam PCOS criteria. We researched the association between FSH total dose and live birth rates (LBRs) using multivariate regression analysis. In addition, a model for nonlinear relationships based on a two-part linear regression was applied. The analysis of threshold effects indicated that LBR increased with every 1000 IU FSH when the concentration of FSH was lower than 1410 IU (OR 1.55, 95% CI [1.05, 2.28]); however, a negative association between FSH dose and LBR (OR 0.94, 95% CI [0.89, 0.99]) was found when the FSH total dose was higher than 1410 IU. It is worth noting that the relationship between LBR and FSH dose varied among patients of different ages (OR 0.92 vs 1.06, P for interaction < 0.05).

## Introduction

In assisted reproductive technology (ART) cycles, ovarian stimulation uses exogenous gonadotropins to induce multiple follicles to develop simultaneously, providing more mature oocytes and enough embryos for transfer^1^. Antral follicle count (AFC) is usually positively correlated with live birth^1–5^. It is known that the total dose of follicle-stimulating hormone (FSH) to stimulate ovarian depends on patient characteristics (e.g. age, ovarian reserve, weight) and responsiveness to FSH^6^. Therefore, patients who respond poorly or slowly need to increase doses^7–10^.

With regard to the role of FSH in in-vitro fertilization (IVF)/intracytoplasmic sperm injection (ICSI), previous research found that there exist a positive dose–response association between pregnancy outcome and FSH. High FSH harvests more mature oocytes and reduces cycle cancellation rates; conversely, in patients predicted to have a hyper-response to FSH, increasing the FSH dose may increase the risk of OHSS and related risk for cycle cancellation^11,12^. However, some studies have found that expected poor response patients can’t get benefit from high FSH^13–17^. Controlled ovarian stimulation (COS) may have adverse influence on embryo quality and may uncouple oocytes from the somatic cells of the follicle, leading to chromosomal abnormalities or interfering with the natural selection of optimal oocytes^18–20^. Studies on cattle have found that high dose of FSH are detrimental to the ovulatory follicular function, reducing the developmental potential of oocytes and leading to a reduced rate of transferable quality embryos^21,22^. One study including > 650,000 ART cycles found that high dose of FSH were adversely associated with live birth rates (LBRs)^23^. A latest meta-analysis did not find that the dose of FSH affected the LBRs or pregnancy rate^9^. A randomized controlled trial of non-PCOS women found no statistically significant difference in cumulative live birth rates between the two groups receiving different FSH doses^24^. However, few studies with adequate sample size have investigated whether FSH applied provides an independent factor affecting LBRs from fresh cycles in non-PCOS women.

Given the contradictory evidence above, there is growing interest in the influence of the dose of FSH on live births. How to determine the dose of FSH during COS is necessary to clinician's judgement. This paper aimed to research the relationship between total FSH dose and live births in the non-PCOS population and to explore whether there is a dose–response inflection point for live births and FSH applied.

## Methods and materials

### Study population and design

**Figure 1:**
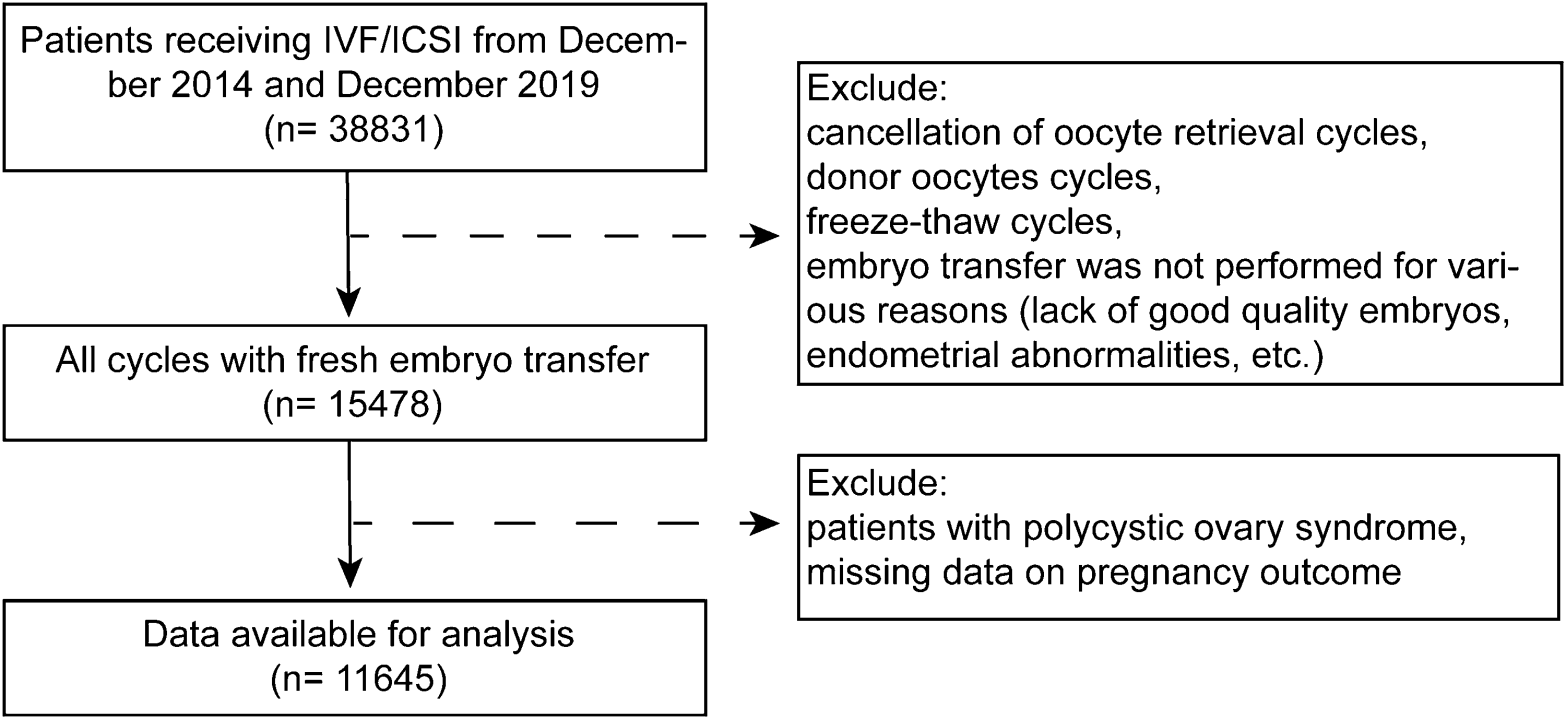
Flow chart for selection of patients from December 2014 to December 2019.

Our study population includes all autologous IVFs between December 2014 and December 2019. Cancellation of oocytes retrieval cycles, donor oocytes cycles, freeze–thaw cycles, embryo transfer was not performed for various reasons (lack of good quality embryos, endometrial abnormalities, etc.), patients with PCOS, and missing data on pregnancy outcome were not included. The detailed flow chart is shown in Fig. 1.

The ovarian stimulation protocol has been introduced previously^25^. In brief, ovarian stimulation in fresh cycles was achieved with combination of gonadotropin-releasing hormone agonist, antagonist and recombinant follicle-stimulating hormone. Serial ultrasound examinations and hormone measurements were performed the ovarian response. All patients received recombinant FSH and/or HMG for COS. The dose of HMG was determined based on multiplying vials number of HMG given by 75 IU and adding this to the total amount of FSH given. When at least 1 follicle ≥ 18 mm in diameter or 3 follicles ≥ 17 mm in diameter were monitored in both ovaries during ovulation promotion, 10,000 units of human chorionic gonadotropin (hCG) injection was given. Oocytes were retrieved under transvaginal ultrasound guidance 36–37 h later. For patients with possible ovarian hyperstimulation syndrome, the embryos are frozen.

Basic demographic characteristics include patient age, BMI, antral follicle count, and type of infertility. Embryo indicators include the number of embryos transferred, stage of embryo culture. Outcome indicators include number of oocytes retrieved, clinical pregnancy rate, and LBRs.

When evaluating the cleavage stage embryos, the selected indicators mainly include the number of blastomeres and the fragmentation rate^26^. Morphological scoring is mainly based on Gardner and Schoolcraft scoring system^27^. Fresh embryo transfer was conducted on the 3rd to 5th day after retrieval. Progesterone gel (Crinone, Merck Serono, Watford, UK) was administrated for luteal phase support starting from the oocyte retrieval day until 14 days after ET.

### Outcome

A positive blood hCG test was performed 14 days after embryo transfer and a positive result was regarded as a biochemical pregnancy. Ultrasound was performed 30 days after embryo transfer and the presence of a gestational sac was regarded as clinical pregnancy. Live birth was defined as the birth of at least one living child.

### Covariates assessment

Covariates were chosen based on clinical experience and studies published in recent years, with the following covariates being included in the model. The continuous variables mainly include female age (in years), infertility duration (in years), body mass index (BMI, kg/m2), and endometrial thickness on the hCG trigger day (mm). The categorical variables mainly included infertility type (primary or secondary infertility), fertilization method (IVF, ICSI or IVF&ICSI), and type of embryo transferred (cleavage stage or blastocyst stage).

### Treatment procedures

Starting in the mid-luteal phase, the patient was given 0.1 mg/day of Decapeptyl (Ferring AG, Dübendorf, Switzerland) as part of the gonadotropin releasing hormone agonist (GnRH-a) protocol. After about 14 days, gonadotropin was given until the hCG trigger day once down-regulation standards (LH < 5 IU/L, E2 < 50 pg/L, FSH < 5 IU/L, thickness of endometrium < 5 mm, follicle diameter and no functional ovarian cyst) were satisfied by serum endocrinology and transvaginal ultrasound examinations. On the second day of the menstrual cycle, patient received 3.75 mg of triptorelin (Ipsen Pharma Biotech, Signes, France) as part of the prolonged GnRH agonist protocol. Once pituitary-ovarian suppression was established, gonadotrophin was administered 28–31 days later. Throughout therapy, the ovarian response was tracked using vaginal ultrasonography and serum hormone levels were measured.

The GnRH antagonist (GnRH-ant) protocol began on day 2 or 3 of menstruation. Daily injections of 0.25 mg Cetrotide (Baxter Oncology GmbH, Frankfurt, Germany) were administered until the hCG trigger day once the leading follicle reached 14 mm diameter.

The injection used as the hCG trigger was either 250 µg of recombinant human follitropin alfa (MerckSerono S.p.A., Geneva, Switzerland) or 8000–10,000 IU of hCG (Lishenbao, Livzon Pharmaceutical Co., Ltd.). The trigger day was the combination of > three follicles with > 17 mm diameter, or > 18 mm diameter in one dominant follicle with acceptable serum hormone levels. On the day of the hCG trigger, patients had blood testing with an empty stomach.

Under the supervision of vaginal ultrasound guidance, posterior fornix puncture and oocyte retrieval were carried out 36–37 h after the hCG triggering.

Blastomere number, degree of fragmentation, embryo symmetry, multinucleation, and degree of cell fusion were used to score the embryos after 72 h. The embryo grading criteria was used to determine which embryos were chosen for transfer^28^. A fresh ET (with no more than two embryos) might be carried out if the endometrium was likewise in good condition (thickness ≥ 8 mm; acceptable morphology) and there were no transfer-related contraindications. On the day of oocyte retrieval, luteal support was initiated with the use of oral contraceptive tablets (10 mg twice daily) and 8% progesterone sustained-release vaginal gel (Xenoto, Merck Serono, Germany), in accordance with consensus^29^. The progesterone and estrogen dosages didn't alter until 14 days after ET, when the blood β-hCG level was measured.

### Statistical analysis

When analyzing the baseline characteristics, the method of mean ± SD is applied to the measurement data, while the method of counting and proportion is applied to the categorical variables. Chi-square tests (for categorical variables), analysis of covariance (normally distributed continuous variables) or a Kruskal–Wallis test (non-normally distributed continuous variables) were used to compare the difference between groups. In this study, we aimed to explore the association between FSH total dose and LBRs based on logistic regression analysis. Crude odds ratios (OR) and adjusted OR with 95% confidence interval (CI) were calculated. Tertiary groups were applied to flexibly model and visualize the association between the FSH total dose and LBR.

In multivariable logistic regression analysis, three models are established: unadjusted crude regression estimation; model I, estimates adjusted for female age and BMI; model II, estimates adjusted for all covariates (female age, infertility duration, BMI, infertility type, stimulation protocol, endometrium thickness on hCG trigger day, embryo stage, and method of fertilization.) (using R packages: doBy, plotrix, stringi, stringr, survival, rms, nnet, car, mgcv, gdata, geepack).

The generalized estimating equation (GEE) framework was used to construct univariable and multivariable regression models for the prediction of live birth. This was done to control for the non-independence of data since some patients contributed more than one cycles in the dataset analyzed. In the process of regression analysis, the result of smooth curve fitting is used to determine whether the independent variable is divided into intervals and general addictive model (GAM) was allied using R packages: doBy, plotrix, stringi, stringr, survival, rms, nnet, car, mgcv, gdata, Hmisc, SparseM, carData, nlme, lattice, Formula, ggplot2. Under the condition of dividing into intervals, each interval is fitted based on the piecewise regression method. When comparing non-segmented and single-segment models, the log-likelihood ratio method is used to determine whether there is a threshold based on the results obtained. According to the model analysis results, the maximum likelihood of the inflection point of the connecting line segment is determined, and the two-step recursive method is applied in the regression (using R packages: doBy, plotrix, stringi, stringr, survival, rms, nnet, car, mgcv, MASS, mgcv, gdata, geepack).

Step 1: Narrow the inflection point to the 10% range of independent variables, and increase it by 5% in the range of 5% to 95%, so that 19 percentage points can be determined, and then set these points as inflection points, so as to obtain the segmented regression model, and compare the results to obtain the maximum likelihood model corresponding to a point. The inflection point is reduced to the ± 4% range of the percentile, and the inflection point determined according to this is marked as Kmin and Kmax.

Step 2: Analyze based on the recursive method to determine the exact inflection point between the two inflection points. The corresponding process is as follows: first, run the model of three inflection points within the two inflection points, and compare the results to determine the maximum likelihood model corresponding to the highest quartile. Then reduce the range of Kmin and Kmax to ± 25% of the corresponding quartile, and carry out iterative operation continuously to finally obtain the specific value of the target variable. Set this point as the inflection point, and the piecewise regression can obtain the optimal result.

Interaction and stratification analysis were conducted based on age, infertility duration, BMI, AFC, endometrial thickness on hCG trigger day, stimulation protocol and method of fertilization (using R packages: doBy, plotrix, stringi, stringr, survival, rms, nnet, car, mgcv, geepack, lmtest).

Data were analyzed with the statistical software packages R (http://www.R-project.org, The R Foundation) and EmpowerStats software (http://www.mpowerstats.com, X&Y Solution, Inc., Boston, MA).

### Statement

The studies involving human participants were reviewed and approved by the ethics committee of the Second Hospital of Hebei Medical University. The patients/participants provided their written informed consent to participate in this study. All methods were carried out in accordance with STROBE guidelines and regulations.

## Results

In this study, 11,645 fresh embryo transfer cycles were analyzed. All cycles were classified into three groups according to the tertile of the FSH dose during ovulation promotion.

### Characteristics of the patients

Baseline characteristics are detailed in Table 1. Women and men in the high FSH dose group were older, with higher BMI, and lower basal AFC. Regarding ovarian stimulation protocols, the prolonged GnRH-agonist protocol was applied more often in women who were given higher FSH doses than those were given lower FSH doses.

**Table 1:** Demographic characteristics of the participants.

Demographic characteristics	FSH total dose applied (IU)			*P* value
	Tertile1: < 2170	Tertile2: 2171–2849	Tertile3: > 2850	
N	3828	3924	3893	
Female age (years)	29.8 ± 4.7	30.7 ± 4.6	31.8 ± 4.8	< 0.001
Male age (years)	30.7 ± 5.2	31.5 ± 5.0	32.4 ± 5.3	< 0.001
Infertility duration (years)	3.0 (2.0–5.0)	3.0 (2.0–5.0)	3.0 (2.0–5.0)	< 0.001
BMI (kg/m^2^)	22.6 ± 3.2	23.2 ± 3.4	24.0 ± 3.8	< 0.001
Basal FSH (mIU/ml)	7.6 ± 3.3	7.9 ± 3.8	8.3 ± 3.7	< 0.001
Basal E2 (pg/ml)	32.0 (16.7–49.0)	31.8 (15.9–48.0)	30.0 (15.0–47.0)	< 0.001
Basal P (ng/ml)	0.6 (0.4–0.9)	0.6 (0.4–0.9)	0.6 (0.3–0.9)	0.337
Basal LH (mIU/ml)	4.3 (3.1–5.8)	4.1 (3.0–5.5)	4.0 (3.0–5.5)	< 0.001
AFC	12.8 ± 5.5	11.7 ± 4.8	11.0 ± 5.0	< 0.001
Endometrium thickness on hCG trigger day (mm)	10.7 ± 2.1	10.9 ± 2.0	10.8 ± 2.1	< 0.001
Infertility type				< 0.001
Primary infertility	2202 (57.5%)	2102 (53.6%)	2024 (52.0%)	
Secondary infertility	1626 (42.5%)	1822 (46.4%)	1869 (48.0%)	
Stimulation protocol				< 0.001
GnRH-agonist protocol	1884 (49.2%)	2023 (51.6%)	1608 (41.3%)	
Prolonged GnRH-agonist protocol	674 (17.6%)	884 (22.5%)	1352 (34.7%)	
GnRH-antagonist protocol	736 (19.2%)	608 (15.5%)	541 (13.9%)	
Data missing	534 (14.0%)	409 (10.4%)	392 (10.1%)	
FSH total dose (IU)	1715.4 ± 369.3	2470.3 ± 187.0	3514.4 ± 582.9	< 0.001
FSH duration (day)	9.0 ± 2.0	10.4 ± 1.4	12.3 ± 1.9	< 0.001
Method of fertilization				< 0.001
IVF	2926 (76.4%)	3137 (80.0%)	3145 (80.8%)	
ICSI	887 (23.2%)	768 (19.6%)	736 (18.9%)	
IVF&ICSI	15 (0.4%)	16 (0.4%)	10 (0.3%)	
Number of oocytes retrieved	10.3 ± 5.6	10.2 ± 5.1	9.3 ± 4.8	< 0.001
Embryo stage				0.032
Cleavage stage	3777 (99.5%)	3879 (99.6%)	3858 (99.8%)	
Blastocyst stage	19 (0.5%)	14 (0.4%)	6 (0.2%)	
Number of embryos transferred				< 0.001
1	367 (9.6%)	274 (7.0%)	131 (2.8%)	
2	3279 (85.7%)	3365 (84.8%)	4273 (91.9%)	
3	182 (4.7%)	285 (7.2%)	246 (5.3%)	

Normal distribution of data was presented as mean ± standard deviation; nonnormal distribution of data was presented as median (interquartile range) and categorical data using number (percentage).

*BMI* body mass index, *FSH* follicle stimulating hormone, *P* progesterone, *E2* estradiol, *LH* luteinizing hormone, *AFC* antral follicular count, *hCG* human chorionic gonadotropin, *GnRH* gonadotropin releasing hormone, *IVF* in vitro fertilization, *ICSI* intracytoplasmic single sperm injection.

Table 2 shows the pregnancy outcomes by FSH dose tertiles divided into three groups. There was obvious difference (P < 0.001) in LBR among three groups, 44.5%, 43.5% and 39.7% respectively. The LBR was obviously lower in group with the highest applied FSH dose than in the reference group (OR 0.82, 95%CI [0.75, 0.90]). Biochemical and clinical pregnancies were also notable lower compared to that of the reference group. Pregnancy loss increased in the tertile 2 and tertile 3 groups than the reference group, but with no significance (P = 0.612).

**Table 2:** Demographic characteristics of the participants stratified by outcome.

Pregnancy outcome	FSH total dose applied (IU)			*P* value
	Tertile1: < 2170	Tertile2: 2171–2849	Tertile3: > 2850	
Live birth				< 0.001
N (%)	1704 (44.5%)	1706 (43.5%)	1546 (39.7%)	
OR (95%CI)	1	0.96 (0.88, 1.05)	0.82 (0.75, 0.90)	
Biochemical pregnancy				< 0.001
N (%)	2120 (56.1%)	2137 (54.9%)	1954 (50.5%)	
OR (95%CI)	1	0.95 (0.87, 1.04)	0.80 (0.73, 0.87)	
Clinical pregnancy				< 0.001
N (%)	1984 (51.8%)	2016 (51.4%)	1860 (47.8%)	
OR (95%CI)	1	0.98 (0.90, 1.07)	0.85 (0.78, 0.93)	
Pregnancy loss				0.612
N (%)	377 (18.1%)	383 (18.5%)	370 (19.3%)	
OR (95%CI)	1	1.02 (0.87, 1.20)	1.08 (0.92, 1.27)	

### Association between FSH dose and LBR

As shown in Table 3, multivariate regression analysis was performed to adjust for possible confounding factors. A negative association was found between FSH total dose and LBR in the crude model with every 1000 IU increase of FSH dosing (OR 0.91, 95% CI [0.87, 0.95]). According to the comparison result, the multivariable regression analyses exhibits no obvious difference after adjusting the factor for female age and BMI in model I (OR 0.97, 95% CI [0.92, 1.01]), and adjusting for female age, infertility duration, BMI, infertility type, stimulation protocol, Endometrium thickness on hCG trigger day, embryo stage and method of fertilization in model II (OR 0.96, 95% CI [0.92, 1.01]).

**Table 3:** Multivariable regression analysis models examining the association between FSH total dose and LBR.

LBR	Crude OR (95%CI)	*P* value	Model I OR (95%CI)	*P* value	Model II OR (95%CI)	*P* value
FSH total dose (1000 IU)	0.91 (0.87, 0.95)	< 0.001	0.97 (0.92, 1.01)	0.155	0.96 (0.92, 1.01)	0.099
Patients were equally divided into three groups according to FSH total dose						
Low	1		1		1	
Intermediate	0.96 (0.88, 1.05)	0.357	1.03 (0.94, 1.13)	0.550	1.01 (0.92, 1.01)	0.914
High	0.82 (0.75, 0.90)	< 0.001	0.94 (0.86, 1.03)	0.201	0.93 (0.84, 1.02)	0.138

Model I: adjusted for female age and BMI. The generalized estimating equation model was applied as the adjusted Model I.

Model II: following factors were adjusted: female age, infertility duration, BMI, infertility type, stimulation protocol, endometrium thickness on hCG trigger day, embryo stage, and method of fertilization. The generalized estimating equation model was applied as the adjusted Model II.

**Figure 2:**
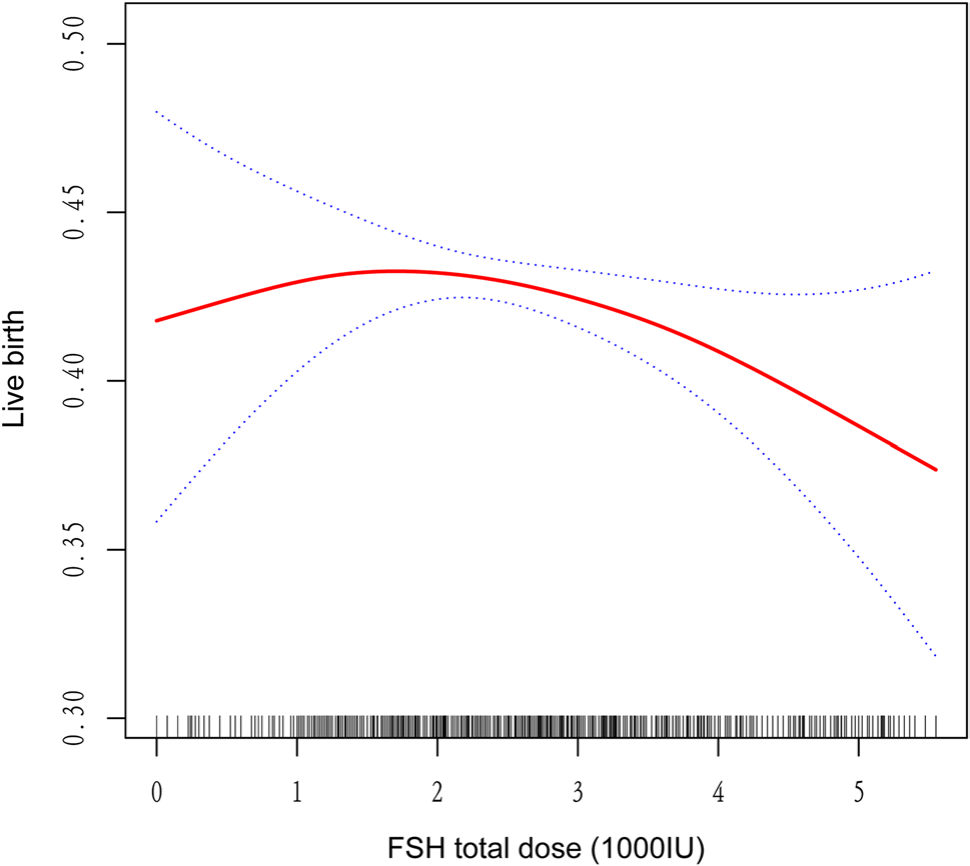
Association between live birth and FSH total dose applied in controlled ovarian stimulation. A threshold, nonlinear association between live birth and FSH total dose was found in a generalized addictive model (GAM). Solid red line represents the smooth curve fit between these two variables. Blue lines represent the 95% of confidence interval from the fit.

However, the smoothing plot demonstrated an obvious relationship between LBR and FSH total dose after adjusting for covariates, including female age, infertility duration, BMI, infertility type, stimulation protocol, Endometrium thickness on hCG trigger day, embryo stage, and method of fertilization. (Fig. 2). With the increase of the FSH dose, the risk of LB increased. When the FSH dose was about 1410 IU, LBR started to decrease (Fig. 2).

In the subsequent study, the two-stage linear regression model was selected for analysis to determine the threshold effect, and the regression results were sorted out as shown in Table 4. The results of this table can be traced, and the inflection point of all variables is 1.41(1000 IU). On the left side of the inflection point, the OR, 95% CI and P values were 1.55, 1.05–2.28 and < 0.05 respectively; On the right side, the three indicators are 0.94, 0.89–0.99 respectively; P < 0.05. We also performed a linear regression model with the result (OR 0.96, 95%CI [0.92–1.01], p > 0.05), indicating that the regression coefficient of FSH total dose on LBR was not significant. The log likelihood ratio check result was 0.013 < 0.05, which reflected that the two-stage linear regression model was better than the one-line linear regression model.

**Table 4:** The result of two-piecewise linear regression model.

Models	Adjusted OR (95%Cl)	*P* value
Model I		
One line slope	0.96 (0.92, 1.01)	0.099
Model II		
Inflection point	1.41	
< 1.41	1.55 (1.05, 2.28)	< 0.05
> 1.41	0.94 (0.89, 0.99)	< 0.05
LRT test	0.013	

Model I, linear analysis; Model II, nonlinear analysis. LRT test, logarithmic likelihood ratio test (p value < 0.05 indicates that Model II is significantly differrent from Model I, which indicates a nonlinear relationship); adjustment variables: female age, BMI, infertility duration, infertility type, endometrium thickness on hCG trigger day, embryo stage, and method of fertilization.

BMI, body mass index; hCG, human chorionic gonadotropin.

Moreover, as shown in Table 5 there was interaction between the female age and FSH total dose (P for interaction < 0.05). Results were different for the patients with more or less than 1 year infertility duration (OR 0.94 vs 1.06, P for interaction = 0.089), although the p-value for interaction was not significant. However, among patients with different BMI (OR 0.94 vs 0.97, P for interaction = 0.247), different AFCs (OR 0.93 vs 1.00, P for interaction = 0.390), different endometrium thickness on hCG trigger day (OR 0.97 vs 0.88, P for interaction = 0.597), primary or secondary infertility (OR 0.96 vs 0.96, P for interaction = 0.322), different protocols (OR 0.86, 0.92 and 0.97, P for interaction = 0.094),and different method of fertilization (OR 0.93 vs 0.97, P for interaction = 0.200), the stratification factors failed to have an interaction effect with FSH total dose in LBR.

**Table 5:** Stratification analysis in different subgroups.

Stratification characteristics	N	LBR		*P* for interaction
		Adjusted OR (95%Cl)	*P* value	
Female age				< 0.05
≤ 35	9756	0.92 (0.87, 0.97)	< 0.001	
> 35	1888	1.06 (0.94, 1.19)	0.351	
Infertility duration (year)				0.089
≤ 1	2239	1.06 (0.95, 1.18)	0.292	
> 1	9224	0.94 (0.89, 0.99)	< 0.05	
BMI (kg/m^2^)				0.247
≤ 24	7247	0.97 (0.92, 1.04)	0.407	
> 24	4248	0.94 (0.87, 1.01)	0.074	
AFC				0.390
≤ 10	4242	1.00 (0.93, 1.09)	0.909	
> 10	5283	0.93 (0.86, 1.00)	0.056	
Endometrium thickness on hCG trigger day (mm)				0.597
≤ 7	442	0.88 (0.70, 1.12)	0.308	
> 7	11,107	0.97 (0.92, 1.01)	0.157	
Infertility type				0.322
Primary infertility	6328	0.96 (0.90, 1.02)	0.167	
Secondary infertility	5317	0.96 (0.89, 1.03)	0.265	
Stimulation protocol				0.094
GnRH-agonist protocol	5515	0.86 (0.80, 0.93)	< 0.001	
Prolonged GnRH-agonist protocol	2910	0.92 (0.84, 1.01)	0.086	
GnRH-antagonist protocol	1885	0.97 (0.85, 1.10)	0.629	
Data missing	1335	0.90 (0.78, 1.05)	0.186	
Method of fertilization				0.200
IVF	9208	0.97 (0.92, 1.02)	0.2233	
ICSI	2391	0.93 (0.83, 1.03)	0.1745	
IVF&ICSI	41	–	–	

The following factors, except the stratification factor itself, were adjusted in the multivariable analysis: female age, BMI, infertility duration, infertility type, endometrium thickness on hCG trigger day, embryo stage, and method of fertilization.

*BMI* body mass index, *AFC* antral follicular count, *Gn* gonadotropin, *P* progesterone, *GnRH* gonadotropin releasing hormone, *IVF* in vitro fertilization, *ICSI* intracytoplasmic single sperm injection.

## Discussion

This paper found that the live birth rate increased and then decreased when total FSH dose increasing during COS and found an inflection point.

In relevant studies, more than two doses of gonadotropins (including hMG) were investigated. As the FSH dose increased, the number of oocytes retrieved increased and the number of available embryos increased^11,12^, explaining the positive correlation in the first part of our study results-curve.

One of the highlights of this article was the joint effect of women age and FSH dose on live birth was significant. It is known that FSH high dose in women with normal ovarian reserve showed no significant differences in fertilization rates, embryo numbers or pregnancy rates between the groups^30–32^. In contrast, for older or low-response patients, it is generally accepted that increasing the FSH dose is an appropriate strategy to improve the number of oocytes retrieved. Our study found a positive correlation between their live birth rate and the total FSH dose applied in women aged > 35 years, which was obviously different from younger patients. This is in accordance with the results of several studies^21,33,34^. However, other studies have concluded that high FSH dose do not overcome the negative effect of increasing age to improve the pregnancy outcome of ART^30,35^. In addition, for the low response population, although a positive correlation was found between LBR and total FSH dose in women in the AFC < 5 group, the results were not statistically significant. The most recent study found that higher dose of FSH has no obvious influence on transferable embryos, pregnancy rates or live birth rates in low response population^10,13,14,17,33,36^. In conclusion, poor ovarian response patients may not benefit from higher FSH doses. In addition, our center did not include records of serum AMH until 2019, as too little data for AMH to provide meaningful reference. Therefore, these predictors of ovarian responsiveness^9,17^ that may be used by physicians to determine dose could not be included in this analysis. We also stratified BMI, AFC, endometrium thickness on hCG trigger day, type of infertility, different protocols, and fertilization method and found that FSH dose was inversely related to LBRs and the results were consistent across stratification.

In the latter part of the curve, the LBRs decreases meaningfully with higher FSH doses, by less than 10%. Our study demonstrated that total FSH doses higher than 1410 IU may be excessive and detrimental to the LBRs. This is in accordance with the growing retrospective correlation studies that also show that high FSH doses are detrimental to IVF success and live birth rates^23,37–39^. Some animal studies showed that high FSH doses may impair oocyte quality and maturation^18–20^. In addition, the adverse effects of exogenous gonadotropins on embryonic development are best demonstrated in rodent models. In vivo and in vitro researches suggested that ovarian stimulation may delay the process of single- or two-celled mouse embryos into blastocysts^40–42^. It has been shown that FSH is important for oocyte-granulosa cell interactions during follicular development^18^. In FSH-deficient female mice, oocyte growth was inefficient, although normal mitochondrial DNA accumulation and Kit ligand-encoding messenger RNA splice forms (designed Kitl1 and Kitl2) were found^42^. FSH regulates connexins, the principal gap junction proteins, and cadherins, the cell–cell attachment proteins in both granulosa cells and oocytes^43^.

However, a comparison between natural cycles and GnRH agonist protocols in infertile women had no differences in oocyte cleavage rates, developmental capacity or embryo fragmentation rates^44^. With regard to embryonic chromosomes, it has been shown that in vitro mature mouse oocytes with high levels of FSH exhibit accelerated nuclear maturation and increased aneuploidy^19^. For human meiotic and mitotic chromosome 21 division errors were also associated with higher FSH doses^45^. Exogenous FSH may increase the aneuploidy rate of embryos^46^. Therefore, we consider that mild stimulation appears to have less impact on the process of nuclear maturation and chromosome segregation, and no adverse effects on pregnancy outcome have been detected^47^. Regarding oocyte development, high doses of exogenous FSH may result in morphological abnormalities in oocytes, such as abnormalities in zona pellucida (ZP), cytoplasm and polar bodies of brown granulosa cells. In contrast, pregnancy rates were lower in brown oocytes, suggesting that brown oocytes affect embryo development^48^. FSH treatment causes transzonal projections (TZPs) to subside, altering oocyte transcriptional activity and meiotic capacity, which may lead to abnormal oocyte development^18^. Moreover, premature luteinization was recorded after patients were given high doses of FSH during ovarian stimulation, leading to significant fluctuations in the follicular microenvironment^49,50^. Therefore, this study found a negative correlation in pregnancy outcome when the total FSH dose exceeded a certain dose. It was also found that frozen embryo transfer reversed the adverse influence of high FSH on LBR in fresh cycles, indirectly suggesting that it may be the endometrium receptivity that is adversely affected by the FSH dose^51,52^. Nevertheless, the mechanisms by which high FSH doses alter follicular development and endometrial tolerance need to be further explored.

It is worth noting that there are three main protocols during ART with varying influence on endogenous gonadotropins. In the prolonged GnRH-agonist protocol, endogenous FSH and LH production is inhibited. Conversely, at the start of the GnRH-agonist protocol, endogenous FSH and LH production is increased. But in the GnRH-antagonist protocol, endogenous FSH and LH is at basal levels. The choice of protocol varies from person to person due to the different response to exogenous gonadotropins and different body weight. However, we did not find a significant difference in the relationship between LBRs and total FSH dose in our stratified analysis and interaction test.

The proper explanation for the relationship between FSH dose applied during COS and LBRs seen in non-randomized researches may associated with patient features such as lower response level to FSH^53^, which may impact LBRs and the dosing of FSH. We were unable to judge whether the dose decision was caused by a previous response to FSH.

This study has some limitations. Firstly, the difference of rFSH and uFSH was not discussed. The relevant meta-analysis contained 14 trials with 1726 women found no difference in pregnancy and LBRs between rFSH and uFSH, nor between the hMG preparation group and the urinary purified FSH group^54^. Given that purified uFSH has partial LH activity but rFSH does not, the confounding factors involved need to be further investigated. Secondly, our study excluded patients with PCOS, a population whose hypersensitive response to exogenous gonadotropins would lead clinicians to individualize treatment^55,56^. Thirdly, this study was retrospective and there were some differences in baseline indicators. Additionally, although some baseline variables were included in the adjustment, the possibility of residual confounding privacy, such as AMH, cannot be ruled out. Hence, it is necessary to obtain high quality randomized controlled trials to explore the associate between total FSH dose applied in COS and LBR. Moreover, this study is a single-center study and more studies from other centers are needed to enhance its credibility and replicability.

## Conclusions

Our results indicated a non-linear association between the total dose of FSH application and LBR. In addition, there was an interaction between women's age and the total dose of FSH application. Therefore, more studies are needed to validate our results and hypotheses and to further investigate the underlying mechanisms.

## Data Availability

The raw data supporting the conclusions of this article will be made available by the authors, without undue reservation. Correspondence and requests for materials should be addressed to NC.

## References

[1] van der Gaast MH, Eijkemans MJ, van der Net JB, de Boer EJ, Burger CW, van Leeuwen FE (2006). Optimum number of oocytes for a successful first IVF treatment cycle. Reprod. Biomed. Online.

[2] Sunkara SK, Rittenberg V, Raine-Fenning N, Bhattacharya S, Zamora J, Coomarasamy A (2011). Association between the number of eggs and live birth in IVF treatment: an analysis of 400 135 treatment cycles. Hum. Reprod..

[3] Fatemi HM, Doody K, Griesinger G, Witjes H, Mannaerts B (2013). High ovarian response does not jeopardize ongoing pregnancy rates and increases cumulative pregnancy rates in a GnRH-antagonist protocol. Hum. Reprod..

[4] Steward RG, Lan L, Shah AA, Yeh JS, Price TM, Goldfarb JM (2014). Oocyte number as a predictor for ovarian hyperstimulation syndrome and live birth: an analysis of 256,381 in vitro fertilization cycles. Fertil. Steril..

[5] Polyzos NP, Drakopoulos P, Parra J, Pellicer A, Santos-Ribeiro S, Tournaye H (2018). Cumulative live birth rates according to the number of oocytes retrieved after the first ovarian stimulation for in vitro fertilization/intracytoplasmic sperm injection: a multicenter multinational analysis including ∼15,000 women. Fertil. Steril..

[6] Huber M, Hadziosmanovic N, Berglund L, Holte J (2013). Using the ovarian sensitivity index to define poor, normal, and high response after controlled ovarian hyperstimulation in the long gonadotropin-releasing hormone-agonist protocol: suggestions for a new principle to solve an old problem. Fertil. Steril..

[7] Land JA, Yarmolinskaya MI, Dumoulin JC, Evers JL (1996). High-dose human menopausal gonadotropin stimulation in poor responders does not improve in vitro fertilization outcome. Fertil. Steril..

[8] Pereira N, Friedman C, Hutchinson AP, Lekovich JP, Elias RT, Rosenwaks Z (2017). Increased odds of live birth in fresh in vitro fertilization cycles with shorter ovarian stimulation. Fertil. Steril..

[9] Lensen SF (2018). Individualised gonadotropin dose selection using markers of ovarian reserve for women undergoing in vitro fertilisation plus intracytoplasmic sperm injection (IVF/ICSI). Cochrane Database Syst. Rev..

[10] Leijdekkers JA, Torrance HL, Schouten NE, van Tilborg TC, Oudshoorn SC, Mol B (2020). Individualized ovarian stimulation in IVF/ICSI treatment: It is time to stop using high FSH doses in predicted low responders. Hum. Reprod..

[11] Hoomans EH, Mulder BB (2002). A group-comparative, randomized, double-blind comparison of the efficacy and efficiency of two fixed daily dose regimens (100- and 200-IU) of recombinant follicle stimulating hormone (rFSH, Puregon) in Asian women undergoing ovarian stimulation for IVF/ICSI. J. Assist. Reprod. Genet.

[12] Eppsteiner EE, Sparks AE, Liu D, Van Voorhis BJ (2014). Change in oocyte yield in repeated in vitro fertilization cycles: effect of ovarian reserve. Fertil. Steril..

[13] Broekmans FJ (2019). Individualization of FSH Doses in Assisted Reproduction: Facts and Fiction. Front. Endocrinol. (Lausanne).

[14] Liu X (2022). Increased versus standard gonadotrophin dosing in predicted poor responders of IVF: An open-label randomized controlled trial. Hum. Reprod..

[15] Liu X (2023). Effect of increased gonadotropin dosing on maternal and neonatal outcomes in predicted poor responders undergoing IVF: follow-up of a randomized trial. Eur. J. Obstet. Gynecol. Reprod. Biol..

[16] Liu X (2023). Who may benefit from an increased gonadotropin dosing in predicted poor responders undergoing IVF/ICSI? A secondary analysis assessing treatment selection markers of a randomized trial. Eur. J. Obstet. Gynecol. Reprod. Biol..

[17] van Tilborg TC (2017). Individualized versus standard FSH dosing in women starting IVF/ICSI: An RCT. Part 1: The predicted poor responder. Hum. Reprod..

[18] Combelles CM, Carabatsos MJ, Kumar TR, Matzuk MM, Albertini DF (2004). Hormonal control of somatic cell oocyte interactions during ovarian follicle development. Mol. Reprod. Dev..

[19] Roberts R, Iatropoulou A, Ciantar D, Stark J, Becker DL, Franks S (2005). Follicle-stimulating hormone affects metaphase I chromosome alignment and increases aneuploidy in mouse oocytes matured in vitro. Biol. Reprod..

[20] Li M, Zhao Y, Zhao CH, Yan J, Yan YL, Rong L (2013). High FSH decreases the developmental potential of mouse oocytes and resulting fertilized embryos, but does not influence offspring physiology and behavior in vitro or in vivo. Hum. Reprod..

[21] Lerner SP, Thayne WV, Baker RD, Henschen T, Meredith S, Inskeep EK (1986). Age, dose of FSH and other factors affecting superovulation in Holstein cows. J. Anim. Sci..

[22] Karl KR, Jimenez-Krassel F, Gibbings E, Ireland J, Clark ZL, Tempelman RJ (2021). Negative impact of high doses of follicle-stimulating hormone during superovulation on the ovulatory follicle function in small ovarian reserve dairy heifers†. Biol. Reprod..

[23] Baker VL, Brown MB, Lube B, Smith GW, Ireland JJ (2015). Gonadotropin dose is negatively correlated with live birth rate: Analysis of more than 650,000 assisted reproductive technology cycles. Fertil. Steril..

[24] Oudshoorn SC (2017). Individualized versus standard FSH dosing in women starting IVF/ICSI: An RCT. Part 2: The predicted hyper responder. Hum. Reprod..

[25] Xu X, Yang A, Han Y, Wang W, Hao G, Cui N (2022). The association between serum estradiol levels on hCG trigger day and live birth rates in non-PCOS patients: A retrospective cohort study. Front. Endocrinol. (Lausanne).

[26] Puissant F, Van Rysselberge M, Barlow P, Deweze J, Leroy F (1987). Embryo scoring as a prognostic tool in IVF treatment. Hum. Reprod..

[27] Gardner DK, Lane M (1997). Culture and selection of viable blastocysts: a feasible proposition for human IVF. Hum. Reprod. Update.

[28] Balaban B (1997). Istanbul consensus workshop on embryo assessment: Proceedings of an expert meeting. Reprod. Biomed. Online.

[29] Penzias AS (2002). Luteal phase support. Fertil. Steril..

[30] Out HJ, Braat DD, Lintsen BM, Gurgan T, Bukulmez O, Gökmen O (2000). Increasing the daily dose of recombinant follicle stimulating hormone (Puregon) does not compensate for the age-related decline in retrievable oocytes after ovarian stimulation. Hum. Reprod..

[31] Yong PY, Brett S, Baird DT, Thong KJ (2003). A prospective randomized clinical trial comparing 150 IU and 225 IU of recombinant follicle-stimulating hormone (Gonal-F*) in a fixed-dose regimen for controlled ovarian stimulation in in vitro fertilization treatment. Fertil. Steril..

[32] Tan SL, Child TJ, Cheung AP, Fluker MR, Yuzpe A, Casper R (2005). A randomized, double-blind, multicenter study comparing a starting dose of 100 IU or 200 IU of recombinant follicle stimulating hormone (Puregon) in women undergoing controlled ovarian hyperstimulation for IVF treatment. J. Assist. Reprod. Genet..

[33] Klinkert ER, Broekmans FJ, Looman CW, Habbema JD, te Velde ER (2005). Expected poor responders on the basis of an antral follicle count do not benefit from a higher starting dose of gonadotrophins in IVF treatment: A randomized controlled trial. Hum. Reprod..

[34] Bernstein LR, Mackenzie A, Durkin K, Kraemer DC, Chaffin CL, Merchenthaler I (2023). Maternal age and gonadotrophin elevation cooperatively decrease viable ovulated oocytes and increase ootoxicity, chromosome-, and spindle-misalignments: '2-Hit' and 'FSH-OoToxicity' mechanisms as new reproductive aging hypotheses. Mol. Hum. Reprod..

[35] Broer SL, van Disseldorp J, Broeze KA, Dolleman M, Opmeer BC, Bossuyt P (2013). Added value of ovarian reserve testing on patient characteristics in the prediction of ovarian response and ongoing pregnancy: an individual patient data approach. Hum. Reprod. Update.

[36] Lekamge DN, Lane M, Gilchrist RB, Tremellen KP (2008). Increased gonadotrophin stimulation does not improve IVF outcomes in patients with predicted poor ovarian reserve. J. Assist. Reprod. Genet..

[37] Hofmann GE, Toner JP, Muasher SJ, Jones GS (1989). High-dose follicle-stimulating hormone (FSH) ovarian stimulation in low-responder patients for in vitro fertilization. J. In Vitro Fert Embryo Transf..

[38] Hock DL, Louie H, Shelden RM, Ananth CV, Kemmann E (1998). The need to step up the gonadotropin dosage in the stimulation phase of IVF treatment predicts a poor outcome. J. Assist. Reprod. Genet..

[39] Shaia KL, Acharya KS, Harris BS, Weber JM, Truong T, Muasher SJ (2020). Total follicle stimulating hormone dose is negatively correlated with live births in a donor/recipient model with fresh transfer: An analysis of 8,627 cycles from the Society for Assisted Reproductive Technology Registry. Fertil. Steril..

[40] Ertzeid G, Storeng R (1992). Adverse effects of gonadotrophin treatment on pre- and postimplantation development in mice. J. Reprod. Fertil..

[41] Van der Auwera I, D'Hooghe T (2001). Superovulation of female mice delays embryonic and fetal development. Hum. Reprod..

[42] Kumar TR (2018). Fshb Knockout Mouse Model, Two Decades Later and Into the Future. Endocrinology.

[43] El-Hayek S, Clarke HJ (2015). Follicle-stimulating hormone increases gap junctional communication between somatic and germ-line follicular compartments during murine oogenesis. Biol. Reprod..

[44] Ziebe S, Bangsbøll S, Schmidt KL, Loft A, Lindhard A, Nyboe AA (2004). Embryo quality in natural versus stimulated IVF cycles. Hum. Reprod..

[45] Katz-Jaffe MG, Trounson AO, Cram DS (2005). Chromosome 21 mosaic human preimplantation embryos predominantly arise from diploid conceptions. Fertil. Steril..

[46] Baart EB, Martini E, Eijkemans MJ, Van Opstal D, Beckers NG, Verhoeff A (2007). Milder ovarian stimulation for in-vitro fertilization reduces aneuploidy in the human preimplantation embryo: A randomized controlled trial. Hum. Reprod..

[47] Verberg MF, Macklon NS, Nargund G, Frydman R, Devroey P, Broekmans FJ (2009). Mild ovarian stimulation for IVF. Hum. Reprod. Update.

[48] Xu H, Deng K, Luo Q, Chen J, Zhang X, Wang X (2016). High serum FSH is associated with brown oocyte formation and a lower pregnacy rate in human IVF parctice. Cell Physiol. Biochem..

[49] Ubaldi F, Camus M, Smitz J, Bennink HC, Van Steirteghem A, Devroey P (1996). Premature luteinization in in vitro fertilization cycles using gonadotropin-releasing hormone agonist (GnRH-a) and recombinant follicle-stimulating hormone (FSH) and GnRH-a and urinary FSH. Fertil. Steril..

[50] Bosch E, Valencia I, Escudero E, Crespo J, Simón C, Remohí J (2003). Premature luteinization during gonadotropin-releasing hormone antagonist cycles and its relationship with in vitro fertilization outcome. Fertil. Steril..

[51] Munch EM, Sparks AE, Zimmerman MB, Van Voorhis BJ, Duran EH (2017). High FSH dosing is associated with reduced live birth rate in fresh but not subsequent frozen embryo transfers. Hum. Reprod..

[52] Grzegorczyk-Martin V, Roset J, Di Pizio P, Fréour T, Barrière P, Pouly JL (2022). Adaptive data-driven models to best predict the likelihood of live birth as the IVF cycle moves on and for each embryo transfer. J. Assist. Reprod. Genet..

[53] Weghofer A, Barad DH, Darmon SK, Kushnir VA, Albertini DF, Gleicher N (2020). The ovarian sensitivity index is predictive of live birth chances after IVF in infertile patients. Hum. Reprod. Open.

[54] Weiss NS, Kostova E, Nahuis M, Mol B, van der Veen F, van Wely M (2019). Gonadotrophins for ovulation induction in women with polycystic ovary syndrome. Cochrane Database Syst. Rev..

[55] Leijdekkers JA, van Tilborg TC, Torrance HL, Oudshoorn SC, Brinkhuis EA, Koks C (2019). Do female age and body weight modify the effect of individualized FSH dosing in IVF/ICSI treatment? A secondary analysis of the OPTIMIST trial. Acta Obstet. Gynecol. Scand..

[56] Fatemi H, Bilger W, Denis D, Griesinger G, La Marca A, Longobardi S (2021). Dose adjustment of follicle-stimulating hormone (FSH) during ovarian stimulation as part of medically-assisted reproduction in clinical studies: A systematic review covering 10 years (2007–2017). Reprod. Biol. Endocrinol..

